# Supplementary material for: Forced choices reveal a trade-off between cognitive effort and physical pain
Source: eLife. 2020 Nov 17;9:e59410. doi: 10.7554/eLife.59410 (PMC7714391; doi:10.7554/eLife.59410)
Supplement: Supplementary file 1. — (A) contains the average performance on the levels of the N-back task. (B) contains the average pain ratings for the thermal stimuli. (C) contains model estimates from the multilevel logistic regression on choices. (D) contains model estimates from the multilevel logistic regression on choices after controlling for trial number. (E) contains model estimates from the multilevel logistic regression on choices after controlling for the choice made on the previous trial. (F) contains model estimates from the multilevel linear regression on choice response times (RTs). (G) contains the heatmaps of the average predicted RTs from the multilevel linear model. (H) contains scatter plots of predicted vs observed RTs for each combination of effort and pain levels. (I) contains the model estimates from multilevel linear regression on choice RTs for the subset of participants who displayed no significant bias towards either choice option. (J) contains the model estimates from the multilevel linear regression on choice RTs after controlling for the difficulty of the choices. (K) contains model estimates from the multilevel linear regression on choice RTs while including a subject-level predictor of a person’s overall probability of choosing the pain option. (L) contains model estimates from the multilevel logistic regression examining the influence of need for cognition. (M) contains model estimates from the multilevel logistic regression examining the influence of pain catastrophizing. (N) contains model estimates from multilevel logistic regressions examining the influence of the other measures of individual differences. (O) contains correlations between the measures of individual differences. [file elife-59410-supp1.docx]

Supplementary Results

| Supplementary File 1A  *Estimated Average Performance for each N-back Level* | | | | | |
| --- | --- | --- | --- | --- | --- |
| *N*-back Level | Mean Performance | SE of mean | df | *t* | *p* |
| 0-back | .97 | 0.014 | 83.24 | 34.47 | < .001 *** |
| 1-back | .91 | 0.011 | 44.46 | 35.70 | < .001 *** |
| 2-back | .84 | 0.011 | 37.86 | 31.09 | < .001 *** |
| 3-back | .78 | 0.013 | 63.21 | 21.83 | < .001 *** |
| 4-back | .71 | 0.016 | 122.58 | 13.38 | < .001 *** |
| *Note*. Mean performance at each level was tested against a null value of .50, representing chance performance; *** *p* < .001; *p*-values were adjusted using Bonferroni correction. | | | | | |

| Supplementary File 1B  *Reported Pain Ratings Compared Against Calibrated Pain Ratings* | | | | | | | |
| --- | --- | --- | --- | --- | --- | --- | --- |
| Calibrated Pain Level | Average Temperature | Reported Pain Intensity | Mean Difference | SE | df | *t* | *p* |
| 10/100 | 46.87 °C | 20.15 | 10.15 | 1.93 | 54.98 | 5.25 | < .001 *** |
| 28/100 | 47.52 °C | 32.91 | 4.91 | 1.74 | 36.55 | 2.82 | .038 * |
| 45/100 | 48.03 °C | 45.67 | 0.67 | 1.78 | 38.33 | 0.38 | > .99 |
| 63/100 | 48.50 °C | 58.43 | −4.57 | 2.04 | 60.95 | 2.25 | .14 |
| 80/100 | 48.79 °C | 71.19 | −8.81 | 2.44 | 101.94 | 3.612 | .002 ** |
| *Note*. The mean difference of reported pain intensity was compared against the calibrated pain amount for each level. SE represents the standard error of the mean difference from the calibrated pain level; * *p* < .05, ** *p* < .01, *** *p* < .001; *p*-values were adjusted using Bonferroni correction. | | | | | | | |

| Supplementary File 1C  *Model Estimates from Full Dataset Multilevel Logistic Regression on Choices* | | | | |
| --- | --- | --- | --- | --- |
| Variable | β | 95% CI | *z* | *p* |
| (Intercept) | −1.84 | [−2.55, −1.18] | 5.28 | < .001 *** |
| *N*-back level | 1.60 | [1.19, 2.04] | 7.34 | < .001 *** |
| Pain level | −1.24 | [−1.49, −1.02] | 10.39 | < .001 *** |
| *N*-back level × Pain level | −0.12 | [−0.25, 0.003] | 2.03 | .042 * |
| *Note*. The estimates for each coefficient are presented in log-odds and are supplemented with bootstrapped 95% confidence intervals (CIs) around the estimate; * *p* < .05, *** *p* < .001. | | | | |

| Supplementary File 1D  *Model Estimates from Multilevel Logistic Regression Controlling for Trial Number* | | | | |
| --- | --- | --- | --- | --- |
| Variable | β | 95% CI | *z* | *P* |
| (Intercept) | −1.53 | [−2.32, −0.91] | 4.58 | < .001 *** |
| *N*-back level | 1.12 | [0.72, 1.78] | 4.48 | < .001 *** |
| Pain level | −1.16 | [−1.57, −0.86] | 6.56 | < .001 *** |
| Trial number | −0.02 | [−0.05, 0.00] | 1.73 | .084 |
| *N*-back level × Pain level | −0.06 | [−0.27, 0.14] | 0.58 | .56 |
| *N*-back × Trial number | 0.02 | [0.01, 0.04] | 4.14 | < .001 *** |
| Pain level × Trial number | −0.01 | [−0.02, 0.01] | 0.98 | .33 |
| *N*-back level × Pain level × Trial number | −0.00 | [−0.01, 0.00] | 1.11 | .27 |
| *Note*. The estimates for each coefficient are presented in log-odds and are supplemented with bootstrapped 95% confidence intervals (CIs) around the estimate. There was a significant interaction of trial number with *N*-back levels indicating that participants were more likely to choose the pain option at higher effort levels over time. This suggests choices were affected by cognitive fatigue over time, but we observed no decline in *N*-back performance over time; *** *p* < .001. | | | | |

| Supplementary File 1E  *Model Estimates from Multilevel Logistic Regression Controlling for Previous Choices* | | | | |
| --- | --- | --- | --- | --- |
| Variable | β | 95% CI | *z* | *P* |
| (Intercept) | −1.68 | [−2.44, −0.96] | 4.59 | < .001 *** |
| *N*-back level | 1.68 | [1.24, 2.16] | 7.36 | < .001 *** |
| Pain level | −1.18 | [−1.44, −0.95] | 9.55 | < .001 *** |
| Previous choice | −0.70 | [−1.13, −0.29] | 3.27 | .001 ** |
| *N*-back level × Pain level | −0.18 | [−0.31, −0.04] | 2.57 | .010 * |
| *N*-back × Previous choice | −0.06 | [−0.38, 0.26] | 0.39 | .70 |
| Pain level × Previous choice | −0.20 | [−0.51, 0.10] | 1.30 | .19 |
| *N*-back level × Pain level × Previous choice | 0.14 | [−0.07, 0.36] | 1.29 | .20 |
| *Note*. The estimates for each coefficient are presented in log-odds and are supplemented with bootstrapped 95% confidence intervals (CIs) around the estimate; *** *p* < .001. | | | | |

| Supplementary File 1F  *Estimates from Model Predicting RTs to Choose Pain and Effort (Full Dataset)* | | | | | |
| --- | --- | --- | --- | --- | --- |
| Variable | β | 95% CI | *t* | df | *p* |
| (Choose Pain Intercept) | 8.05 | [7.93, 8.18] | 126.41 | 39.10 | < .001 *** |
| (Choose Effort intercept) | 7.94 | [7.84, 8.04] | 158.771 | 33.07 | < .001 *** |
| Choose Pain × Trial | −0.00 | [−0.01, −0.00] | 2.43 | 30.70 | .021 * |
| Choose Effort × Trial | −0.01 | [−0.01, −0.01] | 7.16 | 32.43 | < .001 *** |
| Choose Pain × *SV*_effort_ | 0.00 | [−0.04, 0.04] | 0.02 | 25.26 | .99 |
| Choose Effort × *SV*_effort_ | 0.14 | [0.10, 0.19] | 6.73 | 28.66 | < .001 *** |
| Choose Pain × Pain level | 0.14 | [0.10, 0.18] | 6.56 | 23.79 | < .001 *** |
| Choose Effort × Pain level | −0.06 | [−0.08, −0.04] | 5.63 | 27.81 | < .001 *** |
| *Note*. A linear multilevel model was used to predict RTs for both choosing pain and choosing cognitive effort. The estimates for each coefficient are presented in log-units and are supplemented with bootstrapped 95% confidence intervals (CIs) around the estimate; *** *p* < .001. | | | | | |

*Supplementary File 1G*. Averaged heatmaps of the predicted RTs for each choice from the multilevel linear model (left: choosing pain; right: choosing effort).

Low

High

Low

High

Pain Level

*SV*

e

f

f

o

r

t

Choosing Pain

Low

High

Low

High

Pain Level

*SV*

e

f

f

o

r

t

Choosing Effort


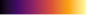


2000

2500

3000

3500

4000

Predicted RT (ms)


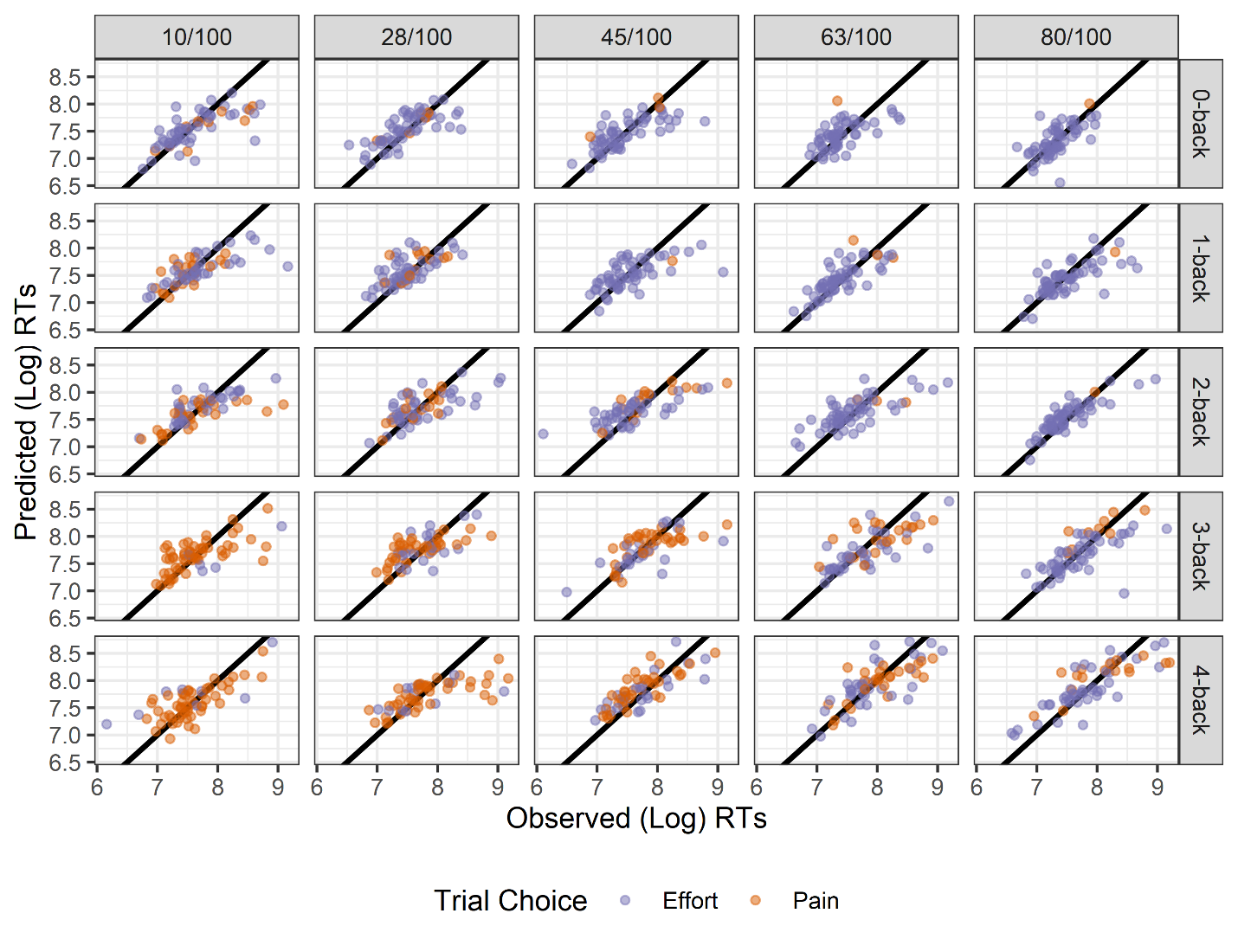
*Supplementary File 1H*. Scatter plots of predicted against observed response times (log-transformed) for each unique combination between pain (columns) and effort (rows) levels for all participants. Points presented in purple represent trials where the effort option was chosen. Points presented in orange represent trials where the pain option was chosen.

| Supplementary File 1I  *Estimates from Model Predicting RTs to Choose Pain and Effort for Participants with 50% Acceptance/Rejection Rate* | | | | | |
| --- | --- | --- | --- | --- | --- |
| Variable | β | 95% CI | *t* | df | *p* |
| (Choose Pain Intercept) | 8.03 | [7.84, 8.23] | 79.08 | 12.05 | < .001 *** |
| (Choose Effort intercept) | 8.05 | [7.84, 8.26] | 73.34 | 12.68 | < .001 *** |
| Choose Pain × Trial | −0.01 | [−0.01, −0.00] | 2.75 | 11.52 | .018 * |
| Choose Effort × Trial | −0.01 | [−0.01, −0.00] | 5.51 | 481.41 | < .001 *** |
| Choose Pain × *SV*_effort_ | −0.03 | [−0.09, 0.03] | 0.95 | 10.30 | .36 |
| Choose Effort × *SV*_effort_ | 0.23 | [0.15, 0.31] | 5.64 | 10.20 | < .001 *** |
| Choose Pain × Pain level | 0.15 | [0.10, 0.21] | 5.13 | 10.34 | < .001 *** |
| Choose Effort × Pain level | −0.05 | [−0.08, −0.02] | 2.88 | 481.87 | .004 ** |
| *Note*. A linear multilevel model was used to predict RTs for both choosing pain and choosing cognitive effort for those participants who displayed an acceptance/rejection rate that was not significantly different from 50% according to a binomial regression fit to each participant’s choices. Data from 11 participants met this criterion. The estimates for each coefficient are presented in log-units and are supplemented with bootstrapped 95% confidence intervals (CIs) around the estimate; * *p* < .05, ** *p* < .01, *** *p* < .001. | | | | | |

| Supplementary File 1J  *Estimates from Model Predicting RTs Controlling for Choice Difficulty* | | | | | |
| --- | --- | --- | --- | --- | --- |
| Variable | β | 95% CI | *t* | df | *p* |
| (Choose Pain Intercept) | 8.21 | [8.09, 8.33] | 130.03 | 64.91 | < .001 *** |
| (Choose Effort intercept) | 8.04 | [7.91, 8.17] | 122.39 | 115.58 | < .001 *** |
| Trial Number | −0.01 | [−0.01, −0.01] | 7.226 | 32.98 | < .001 *** |
| Choose Pain × Indiff. pts. | −0.56 | [−0.85, −0.27] | 3.77 | 937.50 | < .001 *** |
| Choose Effort × Indiff. pts. | −0.35 | [−0.63, −0.08] | 2.53 | 1067.25 | . 012 * |
| Choose Pain × *SV*_effort_ | 0.04 | [0.00, 0.08] | 2.08 | 37.49 | .045 * |
| Choose Effort × *SV*_effort_ | 0.12 | [0.08, 0.16] | 5.77 | 36.17 | < .001 |
| Choose Pain × Pain level | 0.10 | [0.06, 0.15] | 4.63 | 33.52 | < .001 |
| Choose Effort × Pain level | −0.04 | [−0.07, −0.01] | 2.96 | 50.35 | .005 ** |
| *Note*. A linear multilevel model was used to predict RTs for both choosing pain and choosing cognitive effort while covarying out the difficulty of the choices. Because select pain levels appeared on the whole more aversive than cognitive effort, choosing cognitive effort over pain may have been generally easier and potentially faster. We computed the difficulty for each decision by taking the absolute value of the difference between the predicted probability of choosing the pain option and chance probability of choosing pain (50%). These 50% indifference points (“Indiff. pts.”)—i.e., the points where people are expected to choose either option 50% of the time—represent the difficulty of each choice wherein lower values represent difficult choices closer to the 50% indifference point and higher values represent easier choices farther from the indifference points. A linear contrast of the effects of indifference points (i.e., “Choose Pain × Indiff. pts.” vs. “Choose Effort × Indiff. pts.”) revealed no significant difference between the two (*p* = .31). The estimates for each coefficient are presented in log-units and are supplemented with bootstrapped 95% confidence intervals (CIs) around the estimate; * *p* < .05, ** *p* < .01, *** *p* < .001. | | | | | |

| Supplementary File 1K  *Estimates from Model Predicting RTs with Choice Preference as Subject-Level Predictor* | | | | | |
| --- | --- | --- | --- | --- | --- |
| Variable | β | 95% CI | *t* | df | *p* |
| (Choose Pain Intercept) | 7.96 | [7.80, 8.11] | 102.32 | 24.36 | < .001 *** |
| (Choose Effort intercept) | 8.05 | [7.91, 8.20] | 106.73 | 38.95 | < .001 *** |
| Choose Pain × Trial | −0.00 | [−0.01, −0.00] | 2.68 | 30.99 | .012 * |
| Choose Effort × Trial | −0.01 | [−0.01, −0.01] | 7.41 | 32.79 | < .001 *** |
| Choose Pain × P(Pain) | −0.64 | [−1.37, 0.09] | 1.71 | 40.80 | .096 |
| Choose Effort × P(Pain) | 0.53 | [−0.03, 1.07] | 1.88 | 31.72 | .070 |
| Choose Pain × *SV*_effort_ | −0.03 | [−0.07, 0.02] | 1.13 | 15.55 | .27 |
| Choose Effort × *SV*_effort_ | 0.20 | [0.14, 0.27] | 6.13 | 39.19 | < .001 *** |
| Choose Pain × Pain level | 0.14 | [0.09, 0.19] | 5.66 | 13.96 | < .001 *** |
| Choose Effort × Pain level | −0.06 | [−0.10, −0.02] | 3.14 | 44.75 | .003 ** |
| Choose Pain × *SV*_effort_ × P(Pain) | −0.22 | [−0.46, 0.02] | 1.76 | 25.50 | .090 |
| Choose Effort× *SV*_effort_ × P(Pain) | 0.27 | [0.03, 0.51] | 2.24 | 31.31 | .032 * |
| Choose Pain × Pain level × P(Pain) | −0.11 | [−0.41, 0.19] | 0.73 | 25.56 | .47 |
| Choose Effort × Pain level × P(Pain) | 0.02 | [−0.11, 0.15] | 0.26 | 30.35 | .80 |
| *Note*. A linear multilevel model was used to examine whether a decision-maker’s overall preference for pain or effort moderated the effects of pain level and *SV*_effort_ on choice RTs. Here, *P(Pain)*, is a subject-level predictor that represents the overall proportion of pain choices for each individual (centred on 0.5, higher values represent more pain choices). Importantly, higher levels of pain significantly sped RTs to choosing the effort option and this effect was not related to one’s preference for pain or effort. The estimates for each coefficient are presented in log-units and are supplemented with bootstrapped 95% confidence intervals (CIs) around the estimate; * *p* < .05, ** *p* < .01, *** *p* < .001. | | | | | |

| Supplementary File 1L  *Estimates from Model on the Influence of Need for Cognition on Choice Behaviour* | | | | |
| --- | --- | --- | --- | --- |
| Variable | β | 95% CI | *z* | *p* |
| (Intercept) | −1.84 | [−2.54, −1.19] | 5.43 | < .001 *** |
| *N*-back level | 1.60 | [1.19, 2.04] | 7.34 | < .001 *** |
| Pain level | −1.22 | [−1.47, −1.00] | 10.38 | < .001 *** |
| *N*-back level × Pain level | −0.12 | [−0.25, 0.003] | 2.07 | .039 * |
| NFC | −0.38 | [−1.02, 0.26] | 1.20 | .23 |
| *N*-back level × NFC | 0.01 | [−0.43, 0.41] | 0.03 | .98 |
| Pain level × NFC | 0.12 | [−0.07, 0.35] | 1.15 | .25 |
| *N*-back level × Pain level × NFC | −0.12 | [−0.25, −0.01] | 2.12 | .034 * |
| *Note*. The estimates for each coefficient are presented in log-odds and are supplemented with bootstrapped 95% confidence intervals (CIs) around the estimate. The *N*-back level and Pain level predictors were mean centred (i.e., 2-back and 45/100, respectively), and NFC scores represent z-scores across all participants; NFC = need for cognition; * *p* < .05, *** *p* < .001. | | | | |

| Supplementary File 1M  *Estimates from Regression Model on the Influence of Pain Catastrophizing on Choice Behaviour* | | | | |
| --- | --- | --- | --- | --- |
| Variable | β | 95% CI | *z* | *p* |
| (Intercept) | −1.88 | [−2.60, −1.20] | 5.27 | < .001 *** |
| *N*-back level | 1.62 | [1.21, 2.02] | 7.81 | < .001 *** |
| Pain level | −1.25 | [−1.50, −1.02] | 10.35 | < .001 *** |
| *N*-back level × Pain level | −0.11 | [−0.24, 0.02] | 1.88 | .060 |
| PCS | 0.14 | [−0.56, 0.81] | 0.41 | .68 |
| *N*-back level × PCS | −0.48 | [−0.88, −0.10] | 2.42 | .015 * |
| Pain level × PCS | 0.11 | [−0.11, 0.32] | 1.00 | .32 |
| *N*-back level × Pain level × PCS | −0.02 | [−0.13, 0.10] | 0.30 | .76 |
| *Note*. The estimates for each coefficient are presented in log-odds and are supplemented with bootstrapped 95% confidence intervals (CIs) around the estimate. The *N*-back level and Pain level predictors were mean centred (i.e., 2-back and 45/100, respectively), and PCS scores represent z-scores across all participants; PCS = pain catastrophizing; * *p* < .05, *** *p* < .001. | | | | |

| Supplementary File 1N  *Moderating Influences of Individual Difference Measures on Choice Behaviour Effects* | | | |
| --- | --- | --- | --- |
|  | ***N*-back level** | **Pain level** | ***N*-back level**×**Pain level** |
| **NFC** | 0.01 [−0.43, 0.41] | 0.12 [−0.07, 0.35] | **−0.12** [−0.25, −0.01] |
| **PCS** | **−0.48** [−0.87, −0.10] | 0.11 [−0.11, 0.31] | −0.02 [−0.13, 0.10] |
| **FPQ-III** | −0.34 [−0.77, 0.07] | 0.04 [−0.17, 0.27] | −0.09 [−0.21, 0.03] |
| **BAS** | −0.07 [−0.48, 0.35] | 0.05 [−0.16, 0.27] | 0.03 [−0.08, 0.16] |
| **BIS** | 0.30 [−0.10, 0.68] | −0.10 [−0.30, 0.11] | 0.05 [−0.06, 0.17] |
| **OSPAN** | 0.09 [−0.35, 0.49] | −0.02 [−0.21, 0.20] | 0.01 [−0.11, 0.12] |
| **BFI – E** | −0.14 [−0.56, 0.27] | 0.14 [−0.07, 0.35] | −0.07 [−0.19, 0.05] |
| **BFI – A** | 0.35 [−0.07, 0.77] | 0.07 [−0.13, 0.30] | **0.12** [0.004, 0.25] |
| **BFI – C** | 0.18 [−0.22, 0.59] | 0.09 [−0.13, 0.30] | **−0.14** [−0.27, −0.02] |
| **BFI – N** | 0.25 [−0.16, 0.65] | −0.12 [−0.33, 0.10] | 0.06 [−0.06, 0.18] |
| **BFI – O** | −0.14 [−0.54, 0.27] | 0.12 [−0.09, 0.33] | −0.08 [−0.20, 0.04] |
| *Note*. The values here represent the moderating effect of a given individual difference measure on the different effects from the choice behaviour model and are supplemented with bootstrapped 95% confidence intervals (within the square brackets next to each estimate). A negative sign on the interaction term indicates a higher preference for cognitive effort as the levels of pain and *N*-back increase. A positive sign indicates a higher preference for pain at increasing levels of pain and *N*-back; NFC = Need For Cognition Scale (Cacioppo et al., 1984), PCS = Pain Catastrophizing Scale (Sullivan et al., 1995), FPQ-III = Fear of Pain Questionnaire (McNeil & Rainwater, 1998), OSPAN = Operation Span Task (Unsworth et al., 2005), BAS = Behavioral Activation Scale, BIS = Behavioral Inhibition Scale (Carver & White, 1994), BFI – E = Extraversion, BFI – A = Agreeableness, BFI – C = Conscientiousness, BFI – N = Neuroticism, BFI – O = Openness (John et al., 1991). Bolded numbers indicate *p* < .05. | | | |

| Supplementary File 1O  *Correlations between Measures of Individual Differences* | | | | | | | | | | | |
| --- | --- | --- | --- | --- | --- | --- | --- | --- | --- | --- | --- |
|  | **NFC** | **PCS** | **FPQ-III** | **BAS** | **BIS** | **OSPAN** | **BFI – E** | **BFI – A** | **BFI – C** | **BFI – N** | **BFI – O** |
| **NFC** |  |  |  |  |  |  |  |  |  |  |  |
| **PCS** | −.26 |  |  |  |  |  |  |  |  |  |  |
| **FPQ-III** | −.08 | .53 *** |  |  |  |  |  |  |  |  |  |
| **BAS** | .14 | .06 | −.29 |  |  |  |  |  |  |  |  |
| **BIS** | −.31 | .18 | .08 | −.13 |  |  |  |  |  |  |  |
| **OSPAN** | .26 | −.16 | −.16 | −.01 | −.02 |  |  |  |  |  |  |
| **BFI – E** | .20 | .03 | .01 | −.15 | .20 | −.32 |  |  |  |  |  |
| **BFI – A** | .29 | .06 | −.26 | .13 | .27 | .09 | .26 |  |  |  |  |
| **BFI – C** | .57 *** | −.34 * | −.15 | .14 | .02 | −.08 | .15 | .29 |  |  |  |
| **BFI – N** | −.31 | .20 | .03 | −.11 | −.02 | .74 *** | −.43 ** | −.06 | −.02 |  |  |
| **BFI – O** | .57 *** | .04 | < .00 | .07 | .17 | −.35 * | .23 | .01 | .21 | −.23 |  |
| *Note*. The values represent the Pearson correlation coefficient *r* between each individual difference measure. We found no significant correlations between NFC scores and any of the three parameters estimated from the computational model using a parabolic function, *p*s > .23, nor between PCS scores and the *k* or *c* parameters, *p*s > .61. There was a significant negative relationship between PCS scores and *β* parameters, Kendall’s τ_b_ = −.26, *z* = 2.23, *p* = .026, which may suggest less sensitivity to value differences between pain and cognitive effort for those who catastrophize more about pain; NFC = Need For Cognition Scale, PCS = Pain Catastrophizing Scale, FPQ-III = Fear of Pain Questionnaire, OSPAN = Operation Span Task, BAS = Behavioral Activation Scale, BIS = Behavioral Inhibition Scale, BFI – E = Extraversion, BFI – A = Agreeableness, BFI – C = Conscientiousness, BFI – N = Neuroticism, BFI – O = Openness; * *p* < .05, ** *p* < .01, *** *p* < .001. | | | | | | | | | | | |
